# Effects of 12-Week Freestyle Libre 2.0 in Children with Type 1 Diabetes and Elevated HbA1c: A Multicenter Randomized Controlled Trial

**DOI:** 10.1089/dia.2023.0292

**Published:** 2023-11-23

**Authors:** Craig A. Jefferies, Alisa Boucsein, Sara E. Styles, Bronte Chamberlain, Venus R. Michaels, Hamish R. Crockett, Michel De Lange, Anita Lala, Vicki Cunningham, Esko J. Wiltshire, Anna S. Serlachius, Benjamin J. Wheeler

**Affiliations:** ^1^ Starship Child Health, Te Whatu Ora—Health New Zealand, Te Toka Tumai Auckland, Auckland, New Zealand.; ^2^ Liggins Institute and Department of Paediatrics, The University of Auckland, Auckland, New Zealand.; ^3^ Department of Women's and Children's Health, University of Otago, Dunedin, New Zealand.; ^4^ Department of Human Nutrition, University of Otago, Dunedin, New Zealand.; ^5^ Department of Pediatrics, Te Whatu Ora Health New Zealand—Southern, Auckland, New Zealand.; ^6^ Health, Sport and Human Performance, School of Health, University of Waikato, Hamilton, New Zealand.; ^7^ Pacific Edge Ltd., Centre for Innovation, Dunedin, New Zealand.; ^8^ Department of Paediatrics, Te Whatu Ora Health New Zealand—Hauora a Toi, Bay of Plenty, Tauranga, New Zealand.; ^9^ Department of Paediatrics, Te Whatu Ora Health New Zealand New Zealand, Te Tai Tokerau, Whangarei, New Zealand.; ^10^ Department of Paediatrics, Te Whatu Ora Health New Zealand—Capital, Coast and Hutt Valley, Wellington, New Zealand.; ^11^ Department of Paediatrics and Child Health, University of Otago, Wellington, Wellington, New Zealand.; ^12^ Psychological Medicine, The University of Auckland, Auckland, New Zealand.

**Keywords:** children, Intermittently scanned continuous glucose monitoring, Glycemic control, Type 1 diabetes, FreeStyle Libre 2, Self monitoring of capillary blood glucose

## Abstract

**Objective:**

To investigate whether intermittently scanned continuous glucose monitoring (isCGM) reduced glycated hemoglobin (HbA1c) compared with capillary self-monitored capillary blood glucose (SMBG) in children with type 1 diabetes (T1D) and elevated glycemic control.

**Research Design and Methods:**

This multicenter 12-week 1:1 randomized, controlled, parallel-arm trial included 100 participants with established T1D aged 4–13 years (mean 10.9 ± 2.3 years) naive to isCGM and with elevated HbA1c 7.5%–12.2% [58–110 mmol/mol] [mean HbA1c was 9.05 (1.3)%] [75.4 (13.9) mmol/mol]. Participants were allocated to 12-week intervention (isCGM; FreeStyle Libre 2.0; Abbott Diabetes Care, Witney, United Kingdom) (*n* = 49) or control (SMBG; *n* = 51). The primary outcome was the difference in change of HbA1c from baseline to 12 weeks.

**Results:**

There was no evidence of a difference between groups for change in HbA1c at 12 weeks (0.23 [95% confidence interval; CI: −0.21 to 0.67], *P* = 0.3). However, glucose-monitoring frequency increased with isCGM +4.89/day (95% CI 2.97–6.81; *P* < 0.001). Percent time below range (TBR) <3.9 mmol/L (70–180 mg/dL) was reduced with isCGM −6.4% (10.6 to −4.2); *P* < 0.001. There were no differences in within group changes for Parent or Child scores of psychosocial outcomes at 12 weeks.

**Conclusions:**

For children aged 4–13 years with elevated Hba1c isCGM led to improvements in glucose testing frequency and reduced time below range. However, isCGM did not translate into reducing Hba1c or psychosocial outcomes compared to usual care over 12-weeks. The trial is registered within the Australian New Zealand Trial Registry on February 19, 2020 (ACTRN12620000190909p; ANZCTR.org.au) and the World Health Organization International Clinical Trials Registry Platform (Universal Trial Number U1111-1237-0090)

## Introduction

Self-monitoring of glucose levels, whether by self-monitored capillary blood glucose (SMBG), real-time continuous glucose monitoring (rtCGM), or intermittently scanned continuous glucose monitoring [isCGM]), is strongly recommended for children with type 1 diabetes (T1D).^1^ When SMBG measures are used, there is a recommendation to perform 6–10 checks per day; however, it is rare to achieve this.^1,2^ To this end, continuous glucose monitoring (CGM) is now ever increasingly the preferred glucose monitoring technique in current guidelines both in adults and children.^3^ The two related CGM technologies, rtCGM and isCGM, both use a subcutaneously placed sensor to measure interstitial glucose, and both have been shown to offer advantages over SMBG.^4,5^

When compared with first-generation isCGM, rtCGM systems are superior in terms of glucose control, specifically time in range (TIR) and glycated hemoglobin (HbA1c).^6,7^ This may, in part, be due to glucose threshold alerts that were absent from first-generation isCGM systems. Second-generation isCGM is now available, and as well as improved sensor performance offers limited glucose threshold alerts.^8^ These optional glucose threshold alerts may particularly benefit families of children with the above recommended HbA1c, given that the alarms prompt action to prevent and treat both above and below target glucose levels. In adults with out-of-target glucose control, recent data confirm that the isCGM 2.0 system improves glucose control compared with SMBG.^8^ This improves on performance data for the first-generation system, which showed advantages in preventing hypoglycemia, improved glucose testing frequency, but to date no data supporting improvements in glycemic control.^4^

However, in children aged 4–13 years, there are no published randomized controlled trials (RCTs) on either first- or second-generation isCGM. These data are required, as when compared with adult data, pediatric technology outcomes are not always comparable.^9^ We therefore conducted a 1:1 randomized controlled trial in children with elevated glycemic control, comparing second-generation isCGM (Libre 2.0) with SMBG, with the primary outcome being the effectiveness in reducing HbA1c, along with other secondary glucose metrics and psychosocial measures.

## Methods

This 12-week multisite 1: 1 randomized, 2-arm, parallel, controlled open-label study was conducted across five diabetes centers in New Zealand: Northland (NDHB), Auckland (ADHB), Bay of Plenty (BOPDHB), Capital and Coast (CCDHB), and Southern (SDHB) from October 2020 to August 2022. For full details, see the published study protocol.^10^

Eligibility criteria were children aged 4–13 years (inclusive), diabetes duration of ≥6 months, and HbA1c between 7.5% and 12.2% (58–110 mmol/mol) (at time of enrollment and insulin dose >0.5 U/[kg·d]). Exclusion criteria included continuous use of any CGM in the previous 3 months (excluding in-hospital), was defined by use of any CGM for ongoing week-to-week home use in the past 3 months—as opposed to a sensor provided by the clinic to capture 1–2 weeks of data as a one-off in the past 3 months, participation in any other study that could affect measurements, or any severe psychiatric/physical comorbidity, which may have treatment disrupted by agreeing to take part in the trial. Potentially eligible children from the participating sites were invited to participate, and interest was also taken from families at satellite sites following advertising on social media.

Following consent, basic demographic information about children and their parents was collected at the screening visit with outcome measurements assessed at baseline and 12 weeks. HbA1c was measured using a calibrated point-of-care device (DCA Vantage analyzer; Siemens Healthcare Diagnostic Ireland). All consented and eligible participants were asked to wear a blinded sensor (Freestyle Libre Pro; Abbott) for up to 14 days (7 days minimum). While wearing the blinded sensor and before randomization, children and parents were asked to complete age-appropriate psychosocial questionnaires examining quality of life (PedsQL Diabetes Module 3.2 young child/child/teen version, as appropriate), fear of hypoglycemia (Hypoglycemia Fear Survey), and participants aged 10–14 years (inclusive) were also asked to complete the Self Efficacy for Diabetes Self-Management (SEDM).^11–13^ Parents were asked to complete equivalent questionnaires about their children. These surveys were completed again at 12 weeks (see Consort Diagram in Supplementary Material S1).

Data on adverse events, including severe cutaneous reactions and severe hypoglycemia and ketoacidosis were self-reported by phone or text messages, but no further contact was made between research staff and the participants in either group during the study period. All medical advice continued with their usual diabetes service (diabetes clinics are attended regularly [at least every 3 months] by a multidisciplinary team [pediatric endocrinologist/diabetologist/pediatrician, diabetes nurse specialist, dietitian, psychologist]).

The treatment group allocation was revealed at visit 2, which coincided with the end of the 2-week blinded continuous interstitial glucose collection period. Participants were randomized in a 1:1 ratio to either the control (SMBG) group or the intervention (isCGM) group by research staff using a randomization module in REDcap.^14^ Only the biostatistician was masked to group allocation. The intervention consisted of a FreeStyle Libre 2.0 isCGM system (sensors, reader, USB cable, power adapter, user's manual, and quick start guide) and structured education from trained research staff. Education included sensor insertion and interpreting the readings and arrows from the isCGM (see Supplementary Material S2).

The first sensor was applied by research staff. Participants inserted the next sensor 14 days later themselves at home, and for the remainder of the study, and contacted research staff for technical support as required. Participants were instructed to scan a minimum of 6–10 times each day with no longer than 8 h between two scans, but no upper limit was set. The initial recommended reader alert settings were 3.9 mmol/L (70 mg/dL) and 15.0 mmol/L (270 mg/dL). These could be modified as required. As a safety precaution, participants were recommended to perform SMBG to confirm their glucose level before therapeutic interventions for hypoglycemia levels. To prevent sensor loss before the end of the 14-day sensor session, participants were shown examples of cohesive tape to be used to attach the sensor securely in the event the adhesive becomes loose.

Control group participants continued SMBG using conventional finger-stick blood glucose (BG) testing with a glucometer but were recalled at week 10 to be refitted with a second blinded isCGM sensor, which they wore for weeks 10–12 of the study. This was used to compare with the isCGM data from the intervention group for the same 2 weeks.

To maximize study recruitment, all participants received a second-generation isCGM in an open and supported 12-week extension phase after the RCT.

### Ethics

The protocol underwent Māori (indigenous New Zealanders) consultation, which fostered input into this study. The study protocol was approved by the Northern A Health and Disability Ethics Committee (ethics reference: 20/NTA/12). All district health boards approved recruitment and conduct of the study at their site. The isCGM manufacturer was not involved with the planning, funding, or the conduct of the study.

### Statistical analysis and data management

As described in our published methodology, a sample size of 88 (44 participants in each group) was estimated to provide 80% power to detect a difference in changes in HbA1c of 7 mmol/mol (0.75%) between the intervention and control groups using standard deviation (SD) of 15 mmol/mol and correlation of 0.7 between repeated observations on the same person and a two-sided test at the 0.05 level.^10,15^ Randomization was performed within REDCap at each study site as previously outlined.^10^ To account for a small amount of missing data and loss to follow-up, we aimed to recruit at least 100 participants (50 participants per group) at baseline.

The primary analysis followed intention to-treat principles with all participants analyzed in the group to which they were randomized, regardless of actual sensor wear. Additional analyses included standard glycemic metrics, glucose monitoring frequency and adherence, episodes of severe hypoglycemia, episodes of diabetic ketoacidosis (DKA), and psychosocial variables. Mean differences and *P* values were also additionally adjusted for level at baseline and gender. We did not adjust for multiple comparisons, because the various tests are highly nonindependent. All analyses were done with R, version 4.1. A *P*-value of <0.05 was considered significant.

Recruitment was from July 1, 2021, to June 1, 2022, with all subjects completing the 12-week RCT by September 1, 2022.

## Results

Baseline characteristics are presented in Table 1, and CONSORT diagram is shown in Supplementary Figure S2. One hundred children aged 4–13 were enrolled in the study, 49 to isCGM (Libre 2.0) intervention and 51 to SMBG control. The full RCT was completed by 92 children.

**Table 1.** Demographic and Clinical Characteristics of the Participants at Baseline

	All	Intervention (*N* = 49)	Control (*N* = 51)	*P*
Age (years), mean (±SD)	10.88 (2.3)	10.96 (2.39)	10.79 (2.22)	0.72
*n* (% Female)	58 (58%)	33 (67.3%)	25 (50.0%)	0.12
Prioritized ethnicity, *n* (%)^a^				0.56
Māori^b^	25 (25.0)	14 (28.6)	11 (21.1)	
Pacific youth	22 (22.0)	8 (16.3)	14 (28.0)	
New Zealand European	37 (37.0)	19 (38.8)	18 (36.0)	
Asian/other	15 (15.0)	8 (16.0)	7 (14.0)	
NZDep13, *n* (%)^c^				0.63
Quintiles 1–3 (low deprivation)	26 (28.9)	15 (33.3)	11 (24.4)	
Quintiles 4–7 (medium deprivation)	31 (34.4)	14 (31.1)	17 (37.8)	
Quintiles 7–10 (high deprivation)	33 (36.7)	16 (35.6)	17 (37.8)	
BMI *z*-score, mean (±SD)	1.25 (1.11)	1.35 (1.06)	1.14 (1.16)	0.35
Duration of diabetes (years)	4.23 (2.95)	4.4 (2.94)	4.06 (2.98)	0.56
Insulin therapy, *n* (%)				0.39
MDI	83 (83.8)	39 (79.6)	44 (88.0)	
CSII	16 (16.2)	10 (20.4)	6 (12.0)	
Insulin estimated total daily dose (U/[kg·d]), mean (±SD)	0.97 (0.37)	1.03 (0.41)	0.92 (0.33)	0.13
HbA1c (%), mean (±SD)	9.05 (1.26)	8.99 (1.17)	9.11 (1.35)	0.63
HbA1c (mmol/mol), mean (±SD)	75.41 (13.76)	74.73 (12.75)	76.06 (14.76)	0.63
SMBG checks/day, mean (±SD)	4.32 (2.5)	4.71 (2.73)	3.89 (2.17)	0.15

^a^ The participants (or parents/guardians) could select more than one ethnic group. However, they were assigned to a single ethnic group for statistical evaluation with the list prioritized in the standardized order of Māori; Pacific Islander; Asian; Middle Eastern, Latin American, or African; and New Zealand European or other.

^b^ Māori are the indigenous people of New Zealand.

^c^ NZDep13, The New Zealand Deprivation Index is an area-based measure of socioeconomic deprivation (in which 1 represents the least socioeconomic deprivation and 10 the most deprived). Post office box and some rural addresses cannot be derived from this index, and thus, there are NZDep13 scores available for 45/50 (90%) in the intervention group and 45/52 (86.5%) in the control group.

BMI, body mass index; CSII, continuous subcutaneous insulin infusion; HbA1c, glycated hemoglobin; MDI, multiple daily injections; SD, standard deviation; SMBG, Self Monitoring of capillary blood glucose.

### Glycated hemoglobin

As shown in Table 2, at baseline the overall mean (SD) HbA1c was 9.05 (1.26) % [75.41 (13.6) mmol/mol]: the mean HbA1c in the isCGM intervention group was 8.99 (1.17) % [74.73 (12.75) mmol/mol] and 9.11 (1.35) % [76.06 (14.76) mmol/mol] in the SMBG control group. As shown in Table 2, at the end of the 12-week randomized trial, there was no significant between-group difference for change in HbA1c, adjusted difference 0.2% (95% confidence interval [CI]: −0.21 to 0.6) [2.14 mmol/mol (−2.27 to 6.54)], *P* = 0.33.

**Table 2.** Comparison of Glycemic Metrics Between Groups at Baseline and at 12 Weeks

	Baseline		12 Weeks		Difference in unadjusted changes at 12 weeks (95 CI)*^a^*	*P* ^b^
	Intervention	Control	Intervention	Control		
HbA1c^c^						
HbA1c %	9.0 (1.2)	9.1 (1.4)	9.0 (1.2)	9.0 (1.3)	0.23 (−0.21 to 0.67)	0.3
HbA1c (mmol/mol)	74.7 (12.8)	76.1 (14.8)	75 (12.9)	75 (14.6)	2.5 (−2.32 to 7.32)	0.3
Glucose monitoring frequency (daily)^d^						
Interstitial (intervention) versus capillary (control)	4.7 (2.7)	3.9 (2.2)	8.9 (5.1)	3.2 (1.6)	4.89 (2.97 to 6.81)	<0.001)
Interstitial + capillary (intervention) versus capillary (control)	N/A	N/A	10.7 (4.6)	3.2 (1.6)	6.77 (4.81 to 8.72)	<0.001)
Mean glucose mmol/L (SD)	11.2 (2.0)	11.6 (2.7)	12.0 (1.7)	11.5 (2.6)	−1.32 (−2.67 to 0.03)	0.051
Coefficient of variation % (SD)	46.1 (8.5)	46.5 (9.9)	40.8 (4.7)	46.9 (8.5)	2.36 (−2.66 to 7.37)	0.35
TIR data						
% Time in target: 3.9–10 mmol/L (70–180 mg/dL)^e^	28.7 (16.6)	27.3 (16)	36.4 (13.2)	25.1 (13)	8.3 (Int) 3.0 (Control) (CI: −2.7 to 13.4)	0.19
% Time spent: <3.9 mmol/L (<70 mg/dL)^f^	10.7 (11.3)	10 (8.1)	2.9 (2.8)	10.6 (7.5)	−6.4 (Int) 1.0 (Control) (CI: −10.6 to −4.2)	<0.001
% Time spent above 10 mmol/L (180 mg/dL)^g^	60.6 (18.8)	62.2 (20.9)	61.1 (13.3)	64.3 (16.6)	−1.6 (Int) −3.8 (Control) (CI: −6.6 to 11.1)	0.62

Data are mean (±SD) unless otherwise stated.

^a^ *P -*value based on HbA1c measurements recorded in millimoles per mole.

^b^ A negative value for estimated difference means that the reduction is greater in the intervention group. Adjusting for HbA1c, sex, and ethnicity made no meaningful or relevant difference to unadjusted reported outcomes as above.

^c^ *P* values are based on two-sample *t*-tests. A multinominal model, taking into account compositeness of the TIR, TAR, and TBR, gives similar CIs and *P* values.

^d^ Baseline capillary glucose checks from blood glucose meter 14-day summary (intervention group and control group). The 12-week capillary glucose checks were from FreeStyle Libre reader 14-day summary (intervention group) and blood glucose meter 14-day summary (control group). Difference in changes at 3 months was adjusted for sex and baseline HbA1c and incorporated baseline values as repeated measures.

^e^ The time in the target glucose range is the percentage of time with glucose level between 3.9 and 10.0 mmol/L (70–180 mg/dL). All participants before randomization by blinded FSL pro, from FreeStyle 2.0 reader 14-day summary in the intervention group and by blinded FSL pro in the control group.

^f^ Time below 3.9 mmol/mol is the percentage of time with blood glucose levels less than 73.9 mmol/L (70 mg/dL). All participants before randomization by blinded FSL pro, from FreeStyle 2.0 reader 14-day summary in the intervention group and by blinded FSL pro in the control group.

^g^ Time above 10 mmol/mol is the percentage of time with blood glucose levels above 10 mmol/mol 180 mg/dL (3.9 mmol/L). All participants before randomization by blinded FSL pro, from FreeStyle 2.0 reader 14-day summary in the intervention group and by blinded FSL pro in the control group.

TIR, time in range.

### Glucose monitoring

In the isCGM group, there was an increase in mean (SD) glucose checks performed per day from 4.7 (2.7) SMBG at baseline to 10.7 (4.6) combined interstitial and SMBG per day at 12 weeks. In the SMBG group, the baseline glucose checks decreased from 3.9 (2.2)/day to 3.2 (1.6)/day at 12 weeks. This translated to the isCGM group with combined interstitial and SMBG checking over 6 times more/day at +6.77 checks per day (CI: 4.81–8.72), *P* < 0.001. As shown in Table 2, the results are still similar if the isCGM group's SMBG was excluded (between-group difference +4.89 [CI: 2.97–6.81] for interstitial checks on the isCGM compared with the SMBG group, *P* < 0.001).

### Other glucose metrics

**FIG. 1.**
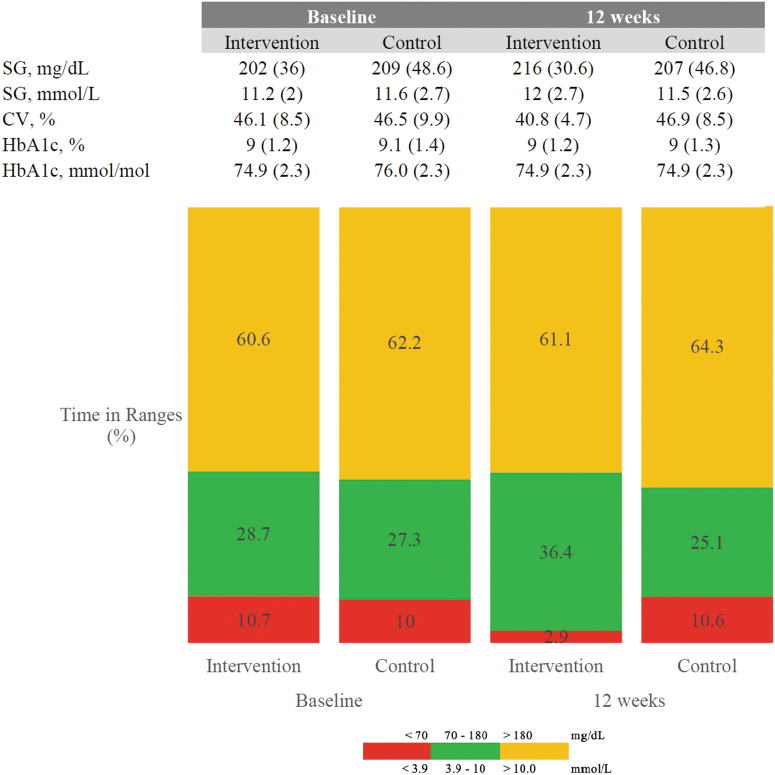
Percentage of time spent in various glucose target ranges at baseline and at 12 weeks in the intervention and control groups. Red is percentage TBR or less than 70 mg/dL (<3.9 mmol/L), green is percentage TIR 70–180 mg/dL (3.9–10.0 mmol/L), and orange is percentage TAR over 180 mg/dL (>10.0 mmol/L). Values shown are mean percentage for the intervention and control groups. TAR, time above range; TBR, time below range; TIR, time in range.

% Time in target 70–180 mg/dL (3.9–10 mmol/L) showed an absolute increase in the isCGM group (28.7% [16.6] at baseline to 36.4% [13.2]) at 12 weeks, and remained similar in the SMBG group (27.3% [16] and 25.1% [13]), however, this did not translate to a statistically significant difference between groups, +4.7% (CI: −2.7 to 13.4), *P* = 0.19 (see Table 2 and Fig. 1).

% Time spent <70 mg/dL (<3.9 mmol/L) showed a difference between groups favoring reduced time <70 mg/dL (<3.9 mmol/L) in the isCGM group (isCGM −6.4% and control +1.0% [CI: −10.6 to −4.2], *P* < 0.001).

% Time above 180 mg/dL (>10 mmol/mol) remained unchanged with no difference between groups at 12 weeks (−1.6% for isCGM and −3.8% for control group, respectively [CI: −6.6 to 11.1, *P* = 0.62]).

There was no difference in mean glucose or glucose Coefficient of Variation percentage between groups at baseline or at 12 weeks.

### Psychosocial assessments

At 12 weeks there was no difference in PedsQL Diabetes Module Total score (or 5 subscales), Fear of Hypoglycemia (2 subscales), nor in Self-efficacy for Diabetes Self-Management score (see Table 3).

**Table 3.** Comparison of Psychological Outcomes Between Groups at Baseline and at 12 Weeks: Children's and Parent's Scores

	Change within intervention group			Change within control group			Difference of change (intervention vs. control)		
	Estimate	95% CI	*P*	Estimate	95% CI	*P*	Estimate	95% CI	*P* ^*^
Children's scores									
PedsQL diabetes									
Diabetes Quality of life	2.08	(−2.04 to 6.2)	0.31	1.86	(−2.37 to 6.08)	0.38	0.23	(−5.58 to 6.03)	0.94
Treatment	3.55	(−3.73 to 10.83)	0.33	3.19	(−3.02 to 9.4)	0.30	0.36	(−9.05 to 9.77)	0.94
Adherence	1.71	(−4.01 to 7.44)	0.55	0.47	(−5.04 to 5.99)	0.86	1.24	(−6.58 to 9.06)	0.75
Worry	−0.01	(−8.27 to 8.26)	0.99	5.56	(−2.5 to 13.62)	0.17	−5.57	(−16.92 to 5.78)	0.33
Communication	5.77	(−2.24 to 13.77)	0.15	4.1	(−5.25 to 13.46)	0.38	1.66	(−10.44 to 13.77)	0.79
Quality of life Total	2.57	(−1.46 to 6.61)	0.2	2.30	(−1.82 to 6.41)	0.26	0.28	(−5.39 to 5.94)	0.92
HFS									
Behavior subscale	0.08	(−0.13 to 0.3)	0.43	−0.02	(−0.23 to 0.18)	0.51	0.11	(−0.19 to 0.4)	0.47
Worry subscale	−0.07	(−0.29 to 0.15)	0.53	−0.09	(−0.36 to 0.18)	0.51	0.02	(−0.33 to 0.36)	0.92
Self-efficiency of diabetes self-management^**^	0.02	(−0.63 to 0.66)	0.96	0.10	(−0.53 to 0.73)	0.75	−0.04	(−0.91 to 0.83)	0.92

	Change within intervention group			Change within control group			Difference of change (intervention vs. control)		
	Estimate	95% CI	*P*	Estimate	95% CI	*P*	Estimate	95% CI	*P* ^*^
Parent's scores									
PedsQL diabetes									
Diabetes Quality of life diabetes	1.74	(−2.04 to 6.2)	0.31	1.86	(−2.37 to 6.08)	0.38	0.23	(−5.58 to 6.03)	0.94
Treatment	1.98	(−3.73 to 10.83)	0.33	3.19	(−3.02 to 9.4)	0.30	0.36	(−9.05 to 9.77)	0.94
Adherence	3.91	(−2.32 to 10.14)	0.21	6.84	(−0.8 to 12.89)	0.028	−2.93	(−11.47 to 5.61)	0.49
Worry	1.75	(−7.61 to 11.1)	0.72	0.55	(−6.47 to 7.58)	0.87	1.19	(−10.33 to 12.72)	0.83
Communication	2.78	(−4.1 to 9.65)	0.42	−2.09	(−9.56 to 5.38)	0.57	4.87	(−5.12 to 14.86)	0.35
Quality of life Total	1.99	(−1.99 to 5.97)	0.32	0.28	(−2.99 to 3.55)	0.86	1.71	(−3.36 to 6.78)	0.51
HFS									
Behavior subscale	0.12	(−0.07 to 0.3)	0.22	−0.04	(−0.17 to 0.1)	0.58	0.15	(−0.07 to 0.38)	0.18
Worry subscale	−0.09	(−0.28 to 0.11)	0.38	−0.23	(−0.39 to 0.08)	0.0033	0.15	(−0.1 to 0.39)	0.23

Data are mean ± SD unless otherwise indicated. PedsQL Diabetes subscale scores range from 0 to 100; higher scores indicate lower problems with diabetes symptoms. PedsQL Treatment subscale scores range from 0 to 100; higher scores indicate lower problems with diabetes-specific barriers. PedsQL Adherence subscale scores range from 0 to 100; higher scores indicate lower problems with adherence. PedsQL Worry subscale scores range from 0 to 100; higher scores indicate lower problems with diabetes-specific worry. PedsQL Communication subscale scores range from 0 to 100; higher scores indicate lower problems with diabetes-specific communication. PedsQL Diabetes Total scores range from 0 to 100; higher scores indicate better diabetes-specific quality of life. Fear of hypoglycemia (HFS) Behavior subscale mean item scores range from 0 to 4; higher scores indicate a greater tendency to avoid hypoglycemia. HFS-Worry subscale scores range from 0 to 4; higher scores indicate more worry concerning episodes of hypoglycemia and its consequences. Self: The SEDM is a 10-item self-report questionnaire for youth aged 10–16 years that examines confidence to carry out self-care behaviors and covers all the key areas of diabetes self-management. Participants are asked “How sure are you that you can do each of the following, almost all the time” and items are rated from 1 (not at all sure) to 10 (completely sure) and averaged.

^*^ Within groups: A positive number means the mean score was higher at week 12.

^**^ For children older than 10 years.

HFS, Hypoglycemia Fear Survey; SEDM, Self-Efficacy for Diabetes Self-Management.

There was no difference in parents' fear of hypoglycemia (Behavior and Worry subscales) at baseline or 12 weeks, and no between-group difference at 12 weeks (*P* = 0.2 for both, see Table 3).

### Adverse events

There was one episode of DKA in the control group and none in the intervention group. There were no episodes of severe hypoglycemia in either group, or any reported hospitalizations for other reasons. There was one case in the intervention group of acquired hyperthyroidism and subsequent drug-induced hepatitis requiring radioactive iodine treatment, resulting in withdrawal from the study.

### Cutaneous reactions

There was one local reaction (erythema, dryness, and irritation) to a baseline blinded Libre pro, data not repeated due to subject choice. There was one reported episode of cutaneous reaction to a skin tape fixation in the intervention group, not repeated once the tape fixation was removed, and no reaction to subsequent sensors. There was one repeated sensor failure in the intervention group (8 sensors “fell off” in the first 4 weeks, and the child discontinued the study). Overall, <1% of Libre 2.0 sensors resulted in a reported cutaneous reaction.

## Discussion

This study investigated second-generation isCGM in children with elevated glycemic control who were naive to any form of CGM. This is the first RCT to be conducted in children aged <13 years worldwide and importantly was carried out independently (i.e., was not industry funded). The main findings are that despite a clear increase in glucose-checking frequency, there were no benefits over 12-weeks seen in the primary outcome HbA1c using isCGM 2.0 compared to SMBG. However, time spent in the hypoglycemic range was considerably improved, and TIR was clinically improved, but this finding did not reach clinical significance.

This lack of improvement (in both the control and intervention groups) in the primary outcome (HbA1c) is consistent with the only other study conducted in pediatric and youth aged children using isCGM (1.0),^4,15^ and with the combined adult and child meta-analysis data on HbA1c and isCGM also, but does differ from a recently published RCT in adults using isCGM 1.0.^5,16^ This null finding for HbA1c is also in contrast to that of the only other randomized control trial of isCGM Libre 2.0 done by the FLASH UK study group, which showed an improvement in HbA1c of mean − 0.5% points after 24 weeks in an adult population with similar baseline HbA1c.^8^ Other than age, ethnicity also differs between these isCGM 2.0 studies with 99% of the UK sample of white European decent compared with ∼50% Māori and Pacific children in our study, including with considerable deprivation.

Similarly, both studies were impacted by COVID effects on procedures and delays in recruitment. Clearly in those struggling with diabetes control and burden, as seen in both pediatric studies to date, sensors alone are less likely to result in substantial improvement in longer term glycemic values as measured by HBA1c.

Interestingly, while HBA1c did not change, there were important glycemic findings when assessing CGM metrics. First, and of considerable clinical relevance, time in hypoglycemia was improved using isCGM 2.0, similar to published studies.^17–19^ Reducing hypoglycemia is an important clinical outcome and of interest to diabetes teams and those living with diabetes. In addition, TIR improved in this study by 8.3%, but this did not reach statistical significance and likely reflects the lack of study power—which was designed for HbA1c. This finding is more consistent with that seen in the FLASH UK study.^8^

In children with T1D and elevated HbA1c, these results will reinforce caution in extrapolating data from adults with T1D.^20^ It may be that in T1D children with elevated HbA1c and already struggling with day-to-day management (consistent with a high % of time in hypoglycemia and hyperglycemia), more advanced technology than isCGM is needed.

Despite the increased use of glucose checking with isCGM, no psychosocial gains were seen on the traditional measures collected.^13,15^ There was minimal if any additional contact with these children above routine care (and in a time of COVID). It may be that the children in our study had more challenges to deal with during this time than diabetes, although recent research suggests that diabetes-related care in teens was relatively protected during COVID.^21,22^

### Strengths and limitations

To our knowledge, this is the only trial to date of isCGM 2.0 in children, and it is important to validate diabetes technology although it is an ever-changing and emerging field.^23^ A key strength is the investigation of isCGM alone, with minimal ancillary support or review. Other strengths include the multisite design of this study, enhancing external validity and helps to have a relative high retention rate despite the challenges of the study's COVID-related time frame. Importantly, these data capture a very ethnically diverse population, with a large percentage with high levels of deprivation and of minority ethnicity. We feel we have added to the literature on the health inequalities of CGM.^24^

The study deliberately targeted those with elevated HbA1c levels who (for whatever reason: cost, social support, or other) have not been on isCGM. Comparatively, they are not the “first movers” in technology, or children/families that are able to exhibit high levels of diabetes management in combination with the challenges of broader socioeconomic deprivation.^25^ Our results are specific to the subgroup of children and families who are not achieving target BG levels and not accessing CGM technology (due to preference or financial or other barriers) and therefore may not be generalized to other groups.

Our age range is both a strength and weakness; from our data, children in this age group are spanning development ages, and very reliant on family structure and support (unlike the studies in adults with T1D). Compared with previous studies on first-generation Libre, we were able to also have the TIR metrics to strengthen this study. LibreView software unfortunately did not provide data on alarm settings and use, and limiting any insights into participant behavior and potential benefits on sensor use or alarms.

A weakness is the ever-changing nature of technological improvement in T1D, and we are aware that at present Libre 2.0 is not the latest version now available, however, much of the world is still using isCGM 2.0, and some locations still 1.0 or are reliant on finger-stick glucose testing. So, this study still has considerable value, especially as the first study of its kind in children, with only one in adults, and also that there are so few worldwide on any form of isCGM. Understanding the evolution of technology and where each type is placed or fits in our management “arsenal” is vital.

### Conclusion

This RCT highlights that isCGM 2.0 is appealing and engages families and children with T1D and elevated HbA1c, as shown by the increased glucose monitoring behavior, and reduced time in hypoglycemia. However, in this study, the use of isCGM (Libre 2.0) did not translate into reducing HbA1c compared with usual care SMBG over a 12-week period. Ongoing efforts to find solutions that improve outcomes both glycemic and psychosocial are required in children experiencing the greatest difficulties and burdens with their diabetes.

## Supplementary Material

Supplemental data

Supplemental data
