# Supplementary figures and images for: Effects of 12-Week Freestyle Libre 2.0 in Children with Type 1 Diabetes and Elevated HbA1c: A Multicenter Randomized Controlled Trial
Source: Diabetes Technol Ther. 2023 Nov 23;25(12):827–35. doi: 10.1089/dia.2023.0292 (PMC10698781; doi:10.1089/dia.2023.0292)

**Figure 1: Consolidated Standards of Reporting Trials (Consort) participant flow diagram.**

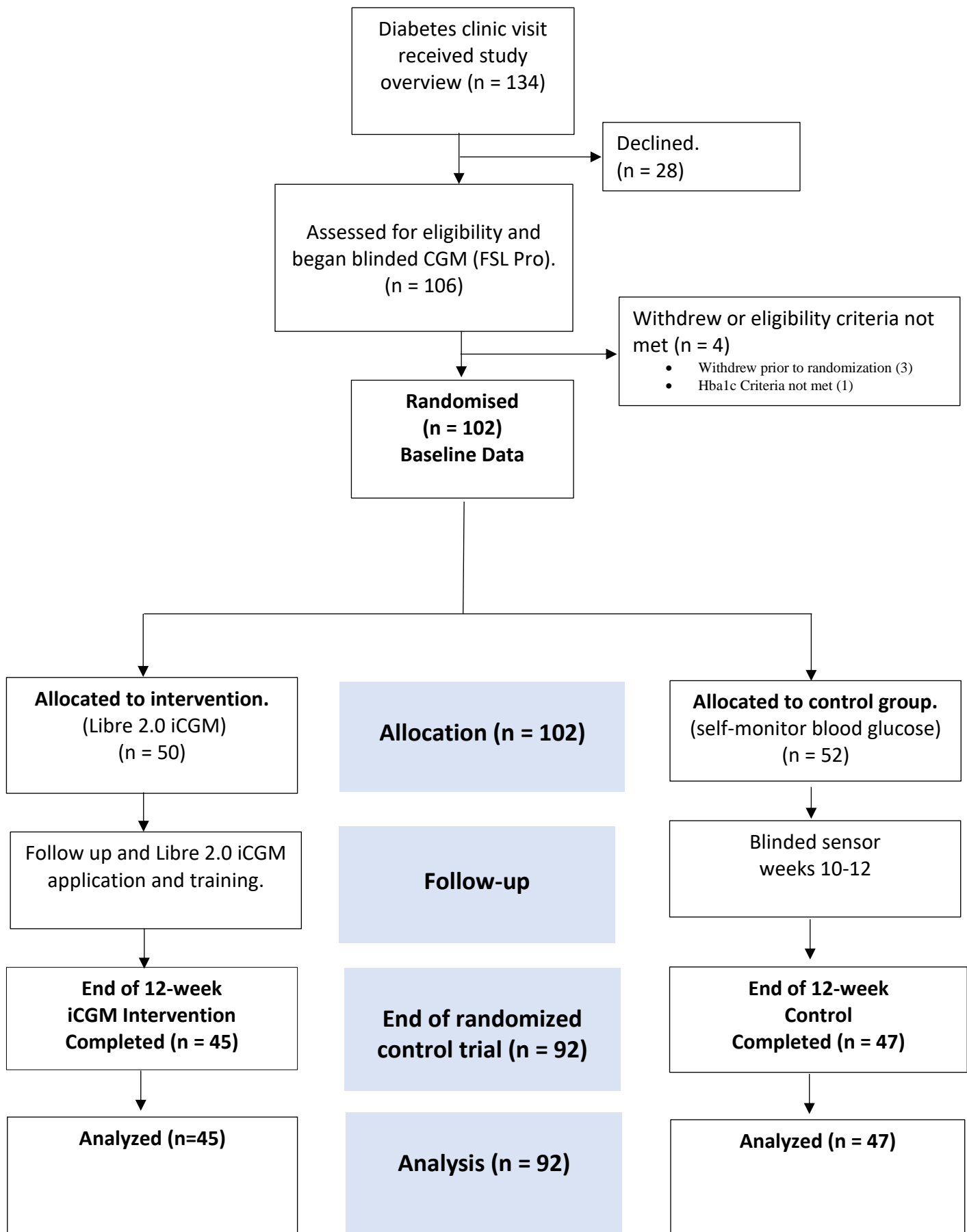

Supplement: Supplemental data [file Supp_DataS1.pdf]
