# Supplementary material for: Effects of 12-Week Freestyle Libre 2.0 in Children with Type 1 Diabetes and Elevated HbA1c: A Multicenter Randomized Controlled Trial
Source: Diabetes Technol Ther. 2023 Nov 23;25(12):827–35. doi: 10.1089/dia.2023.0292 (PMC10698781; doi:10.1089/dia.2023.0292)
Supplement: Supplemental data [file Supp_DataS2.pdf]

FREESTYLE LIBRE FLASH GLUCOSE MONITORING (FGM) SYSTEM

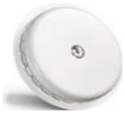

Sensor

- Measures glucose levels in the tissues under your skin
- Worn on upper outer arm
- Each sensor lasts up to 14 days
- Insert each new sensor in a different position to the last. Alternate arms to prevent skin problems. Make sure skin is clean and dry prior to insertion.
- It takes 1 hour for the sensor to warm up after insertion. If you need to know what your glucose level is during this time you will need to do a finger prick glucose.
- Can be worn when showering or swimming
  - Is water-resistant in up to 1 metre of water
  - Don’t immerse in water for longer than 30 minutes
- Don’t wear sensor during an x-ray, CT or MRI scan.
- Sweat during intense exercise may cause the sensor to loosen and need to be replaced
- Use Tegaderm, Tubigrip or K tape to protect your sensor. Cut a hole in the tape overlying the sensor vent. The vent allows moisture to escape and shouldn’t be covered
- Some people experience skin reactions to the sensor or its adhesive. If this happens take a photo of the skin reaction and send it to the research team.

Reader

- The reader is a hand-held touch screen device
- Glucose levels are not constantly shown on the reader. You need to scan the reader over the sensor to transmit data from the sensor to the reader
- The reader has to be within 4cm of the sensor when scanning
- You can scan through clothing with thickness up to 4mm
- After scanning over the sensor the screen on the reader will show
  - Current glucose level
  - A trend arrow
  - Graph of glucose level trends over previous 8 hours

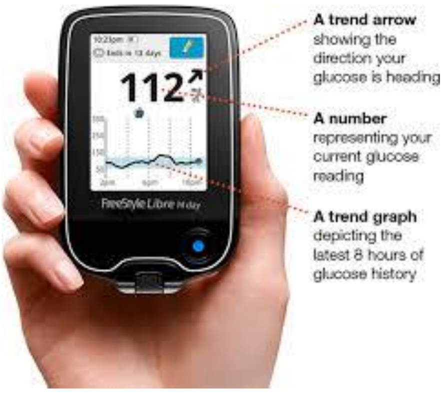

- The reader can store up to 90 days of glucose data

TARGET GLUCOSE RANGE

- 3.9 – 10 mmol/L (this range can be set on your Libre reader)

ALARMS

- This device has an in-built alarm that will alarm if your glucose level is low or high
- It won't however automatically tell you what the glucose level is when it alarms, you need to scan the sensor to see what the glucose level is. You may then need to follow this up with a finger prick glucose check (see below)

FREQUENCY OF SCANNING

- As a minimum you should scan the sensor to check your glucose levels
    - Before meals
    - Before bed
    - Before and after exercise

- You can scan the sensor as often as you like but you must scan at least **6 - 10 times every day**. There should be no longer than 8 hours between two scans including the last scan at night and first scan the following morning

WHEN TO DO FINGER PRICK GLUCOSE CHECKS

- If glucose level on Libre reader is ≤ 4 mmol/L or ≥ 14 mmol/L. This is to confirm what your blood glucose level is and if action is required e.g. hypo treatment or extra-insulin
- If glucose level on Libre reader is rising or falling quickly i.e. trend arrow is going straight up or straight down
- Before giving any therapy e.g. hypo treatment or extra-insulin
    - e.g. when glucose level < 4 mmol/L on Libre reader OR having symptoms of a hypo check finger prick blood glucose level. If finger prick blood glucose level < 4 mmol/L then give hypo treatment.
- If you have symptoms that may be due to low or high blood glucose
- If you have symptoms that don't match the glucose level on the Libre reader
- If you suspect the glucose level on the reader may be inaccurate for any reason
- If your finger prick glucose is ≥ 15 mmol/L or you feel unwell check ketones
- Compression of sensor e.g. lying on the arm your sensor is in when you are asleep can cause the Libre system to give falsely low glucose levels. If in doubt, check a finger prick glucose to confirm your blood glucose level and whether hypo treatment is needed
- Keep yourself hydrated. Dehydration can cause the Libre system to give inaccurate glucose levels

HOW TO INTERPRET TREND ARROWS ON LIBRE READER

- Trend arrows show which way and how quickly your glucose level is changing

Arrow on Reader	Glucose Direction	Predicted Change in 10 minutes
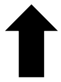	Glucose rising quickly	Rise by more than 1 mmol/L
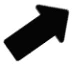	Glucose rising	Rise by 0.6 – 1 mmol/L
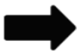	Glucose steady and changing slowly	Rise/fall less than 0.6 mmol/L
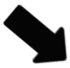	Glucose falling	Fall by 0.6 – 1 mmol/L
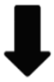	Glucose falling quickly	Fall by more than 1 mmol/L

**Please contact your local diabetes team if you are unsure what to do with your insulin doses*

WHAT TO DO IF MY SENSOR OR READER STOP WORKING

- Contact the research team for advice if your reader or sensor stop working

FURTHER LIBRE INFORMATION RESOURCES AVAILABLE FROM

https://iray.campaign-view.com/ua/viewinbrowser?od=27218d28c96aa859e9afe3a6a54f72fbe1185630859ca1fd0&rd=17d82f84a6156306&sd=17d82f84a615621f&n=11699e4c0f9a4b5&mrd=17d82f84a615620d&m=1
